# Noninvasive assessment for acute allograft rejection in a rat lung transplantation model

**DOI:** 10.14814/phy2.12244

**Published:** 2014-12-18

**Authors:** Ayuko Takahashi, Hiroshi Hamakawa, Hiroaki Sakai, Xiangdong Zhao, Fengshi Chen, Takuji Fujinaga, Tsuyoshi Shoji, Toru Bando, Hiromi Wada, Hiroshi Date

**Affiliations:** 1 Department of Thoracic Surgery, Graduate School of Medicine, Kyoto University, Kyoto, Japan; 2 Department of Thoracic Surgery, Kobe City Medical Center General Hospital, Hyogo, Japan; 3 Department of Surgery, Graduate school of Medicine, Kyoto University, Kyoto, Japan

**Keywords:** Acute allograft rejection, lung impedance, lung transplantation, rejection grade

## Abstract

After lung transplantation, early detection of acute allograft rejection is important not only for timely and optimal treatment, but also for the prediction of chronic rejection which is a major cause of late death. Many biological and immunological approaches have been developed to detect acute rejection; however, it is not well known whether lung mechanics correlate with disease severity, especially with pathological rejection grade. In this study, we examined the relationship between lung mechanics and rejection grade development in a rat acute rejection model using the forced oscillation technique, which provides noninvasive assessment of lung function. To this end, we assessed lung resistance and elastance (*R*_L_ and *E*_L_) from implanted left lung of these animals. The perivascular/interstitial component of rejection severity grade (A‐grade) was also quantified from histological images using tissue fraction (TF; tissue + cell infiltration area/total area). We found that TF, *R*_L_, and *E*_L_ increased according to A‐grade. There was a strong positive correlation between *E*_L_ at the lowest frequency (*E*_low_; *E*_L_ at 0.5 Hz) and TF (*r*^2^ = 0.930). Furthermore, the absolute difference between maximum value of *E*_L_ (*E*_max_) and *E*_low_ (*E*_het_; *E*_max_ − *E*_low_) showed the strong relationship with standard deviation of TF (*r*^2^ = 0.709), and A‐grade (Spearman's correlation coefficients; *r*_*s*_ = 0.964, *P *< 0.0001). Our results suggest that the dynamic elastance as well as its frequency dependence have the ability to predict A‐grade. These indexes should prove useful for noninvasive detection and monitoring the progression of disease in acute rejection.

## Abstract (precis)

After lung transplantation, early detection of acute allograft rejection is important for both in timely treatment and prediction of chronic rejection which is a major cause of late death. We examined the relationship between lung mechanics and rejection grade development in a rat acute rejection model using the forced oscillation technique, which provides noninvasive assessment of lung function. Our results suggest that the dynamic elastance as well as its frequency dependence reflect the perivascular‐interstitial component of rejection severity grade (A‐grade), and this method should prove useful for noninvasive detection and monitoring the progression of disease in acute rejection.

## Introduction

Lung transplantation is a life‐saving procedure for end‐stage lung disease (Christie et al. 2011). However, 5‐year survival rate is 53% (Christie et al. 2011) which is lower than the other organ transplantations. The majority of late death after lung transplantation is due to chronic rejection, manifesting as bronchiolitis obliterans syndrome (BOS). While multiple potential risk factors for the development of chronic rejection have been reported (Lama 2009), acute allograft rejection (AR) is one of the most important risk factors. Therefore, early detection of any abnormality is vital for optimal treatment, and could also contribute to an improvement in the evaluation of chronic rejection.

The histological assessment with multiple transbronchial lung biopsies (TBLB) is still the gold standard for AR diagnosis (Yousem et al. 1996; Wallace et al. 2003), even though this method is invasive and has sampling errors and variability in the pathologic interpretation (Burton et al. 2008; McWilliams et al. 2008; Patil et al. 2008; Arcasoy et al. 2011). While alternative diagnoses have been suggested with immunological and biochemical methods (Aharinejad et al. 2004; Murata et al. 2008; Westall et al. 2008), little attention has been paid to dynamic lung mechanics. Several clinical studies have reported that the forced expiratory volume in 1‐sec (FEV_1_) decreased during AR (Otulana et al. 1990; Van Muylem et al. 1997). However, its detection sensitivity is only 60% in mild or moderate AR (Van Muylem et al. 1997). Although FEV_1_ has been well studied and widely used in various lung diseases, this index is a polyvalent measure that is influenced by various factors (Mead 1979) and hence it is difficult to link to structure.

The forced oscillation technique (FOT) was introduced by Dubois et al. (1956), as a method of characterizing respiratory impedance. The real part of the impedance describes the dissipative mechanical properties of the respiratory system, whereas the imaginary part is determined jointly by the elastic properties that are related to the energy storage capacity and inertial properties. Recently, FOT has been applied to both human subjects (Gillis and Lutchen 1999; Kaczka et al. 2001; Lutchen et al. 2001) and animal models (Lutchen and Gillis 1997; Ito et al. 2004; Bellardine et al. 2005, 2006) of various lung diseases. Some of the advantages in clinical settings include its noninvasiveness with minimal subject cooperation, and its potential ability to detect the heterogeneous distribution of airway and tissue disease from the frequency‐dependent features of the impedance. Although the possibility of early AR detection after human lung transplantation has been reported using FOT (Goldman et al. 2005; Hamakawa et al. 2010), the relationship between histopathology and lung impedance spectra has not been studied.

We hypothesized that the frequency‐dependent characteristics of lung impedance might be correlated with AR grade. To test this hypothesis, we determined the relationship between dynamic lung impedance parameters and histopathological AR grades in a rat model of lung transplantation. Furthermore, because recruitment of distal lung legions influence lung mechanics both in the normal and diseased lung (Bellardine et al. 2005; Kaczka et al. 2005, 2009; Bellardine Black et al. 2007), we also investigated the impact of positive end‐expiratory pressure (PEEP) on the dynamic lung impedance parameters in order to determine the role of recruitment in tissue distortion and heterogeneity for different AR grade levels.

## Materials and Methods

### Animal preparation

The Animal Care Committee of Kyoto University approved all procedures. Thirty‐five male specific pathogen‐free Brown‐Norway (BN; *RT‐1*^*n*^) rats and 17 Lewis (LEW; *RT‐1*^*1*^) rats weighing 280–320 g were enrolled (CLEA Japan Inc., Shizuoka, Japan). Six BN rats served as native controls without transplantation (native group, *n* = 6). All donor lungs were from BN rats. Orthotopic left lung transplantations were performed between BN and BN (isograft group, *n* = 6), and between BN and LEW (allograft group, *n* = 17), using a modified cuff technique (Mizuta et al. 1989). BN to LEW combination was used as a model of acute allograft rejection (AR), which usually developed to total rejection within 7 days. Recipient animals were randomly assigned to be sacrificed at 2, 4, or 6 days after transplantation. The allograft group was subdivided according to AR grade after confirming pathological diagnosis (see Histopathology and Results).

### Left lung mechanics

The animals were anesthetized, tracheostomized, and ventilated with a computer‐controlled small animal ventilator (flexiVent, SCIREQ, Montreal, QC, Canada). To measure left lung impedance (*Z*_*L*_), euthanasia was performed, the right main bronchus was clamped with a steel clip in the open‐chest condition, and ventilation setting was changed to tidal volume of 3 mL/kg and 70 breaths/min of respiratory rate at five different PEEP levels. After 3 min stabilization to adjust for each PEEP level, *Z*_*L*_ was measured using FOT (Dubois et al. 1956; Oostveen et al. 2003) immediately following two recruitment maneuvers with 30 cmH_2_O airway pressure. The forced oscillatory signal was composed of 19 mutually prime sinusoidals between 0.5 and 19.625 Hz and was delivered during an 8‐sec pause of regular ventilation. Peak‐to‐peak amplitude corresponded to the tidal volume.

### Impedance data analysis

The frequency dependence of the real and imaginary part (*R*_L_ and *X*_*L*_, respectively) was obtained from *Z*_*L*_. The lung elastance (*E*_L_) was calculated using the following equation as *E*_L_ = ‐ *ωX*_*L*_, where *ω* = 2*πf*, and *f* is the oscillation frequency. In addition, we extracted the following parameters from the impedance spectra; *R*_L_ at the highest frequencies (*R*_high_), *E*_L_ at the lowest frequency (*E*_low_; *E*_L_ at 0.5 Hz), the highest absolute value of *E*_L_ (*E*_max_), and the absolute difference between *E*_low_ and *E*_max_ (*E*_het_; *E*_max_* − E*_low_) (Lutchen et al. 2001; Bellardine et al. 2005, 2006).

### Tissue processing

After measuring *Z*_*L*_, lungs were extracted and fixed at an airway pressure of 25 cmH_2_O with buffered formalin. Formalin‐fixed lungs were embedded in paraffin and 5 *μ*m in thickness sections and each section was separated at least 100 *μ*m. The hematoxylin and eosin staining was performed for histopathological diagnosis of AR grading and morphometry.

### Histopathology

The pathologist had no knowledge of the specific protocol, and specimens were evaluated according to the Yousem classification (Yousem et al. 1996). In this study, AR was scored based on A‐grade, in which the severity of the perivascular/interstitial component of AR is categorized by the A‐grade that ranges between A0 (no rejection) and A4 (severe rejection) (Table 1).

**Table 1.** Pathological A‐grade classification of acute lung rejection

Grade	Meaning	Appearance
A0	None	No significant abnormality in lung parenchyma
A1	Minimal	Inconspicuous small mononuclear perivascular infiltrate
A2	Mild	More frequent perivascular infiltrates
A3	Moderate	Dense perivascular infiltrates, extension into interstitial and alveolar space, can involve endothelialitis
A4	Severe	Diffuse perivascular, interstitial, and airspace infiltrates with alveolar pneumocyte damage, potential parenchyma necrosis, infarction, or necrotizing vasculitis

### Morphometry

The allograft group was subdivided according to AR grade after histopathological diagnosis. Lung sections were randomly selected and at least 15 regions from each animal photographed. To better quantify the A‐grade from pathological specimens, we calculated tissue fraction (TF) as the ratio of the alveolar wall and cell infiltration areas in a region to the total area of the region using ImageJ software (National Institutes of Health, Bethesda, MD). The original image was converted to a binary image, with black representing the tissue and infiltrating cells, and white representing the airspace. TF was calculated as the ratio of the sum of the black areas within a region to the total area of region (Ito et al. 2004).

### Statistical analysis

All data are presented as mean ± standard deviation (SD). Two‐way ANOVA with Bonferroni was used to evaluate differences among rejection grades and frequencies, and 2‐way repeated ANOVA with Bonferroni was for grades and PEEP levels. One‐way ANOVA with Turkey‐Kramer's post hoc test was used for difference in TF among groups. Pearson's correlation coefficient and Spearman's rank correlation coefficient were used for the association between rejection grade and *E*_het_. Univariate (simple) linear regression analysis was used for the association between TF and *E*_low_, and between SD of TF and *E*_het_. Prism 5.0d (GraphPad Software, Inc. San Diego, CA) and the statistical package R (http://www.r-project.org/) were used for analysis. A significant difference was defined as *P *< 0.05.

## Results

**Figure 1.**
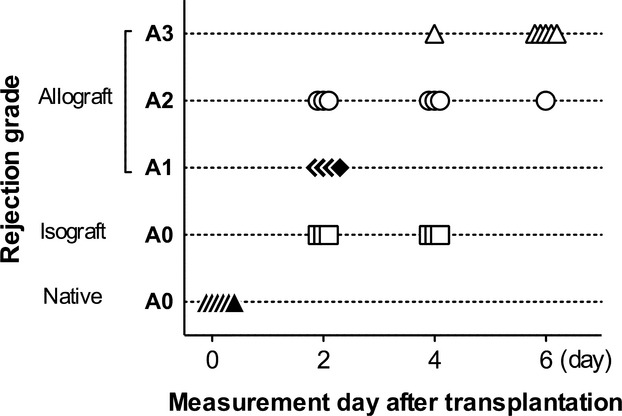
Histopathological rejection A‐grade and measurement day after transplantation. Native control (closed triangle, *n* = 6) and isograft group (open squares, *n* = 6) had no rejection (A0). An allograft group was split into A1 (closed diamonds, *n* = 4), A2 (opened circles, *n* = 7), and A3 (opened triangle, *n* = 6).

The distribution of A‐grade as a function of time in terms of measurement day after lung transplantation is shown in Figure 1. Both the native control (*n* = 6) and the isograft (*n* = 6) groups showed no rejection region (A0). The allograft group (*n* = 17) was subdivided into three groups according to A‐grade, from A1 to A3. There was no A4 region included in our study, because the single lung *Z*_*L*_ could not be measured due to their extremely high airway pressure that often resulted in pneumothorax. Although A1 (*n* = 4) and A3 (*n* = 6) appeared mainly at 2 or 6 days after transplantation, A2 (*n* = 7) was widely distributed between 2 and 6 days.

**Figure 2.**
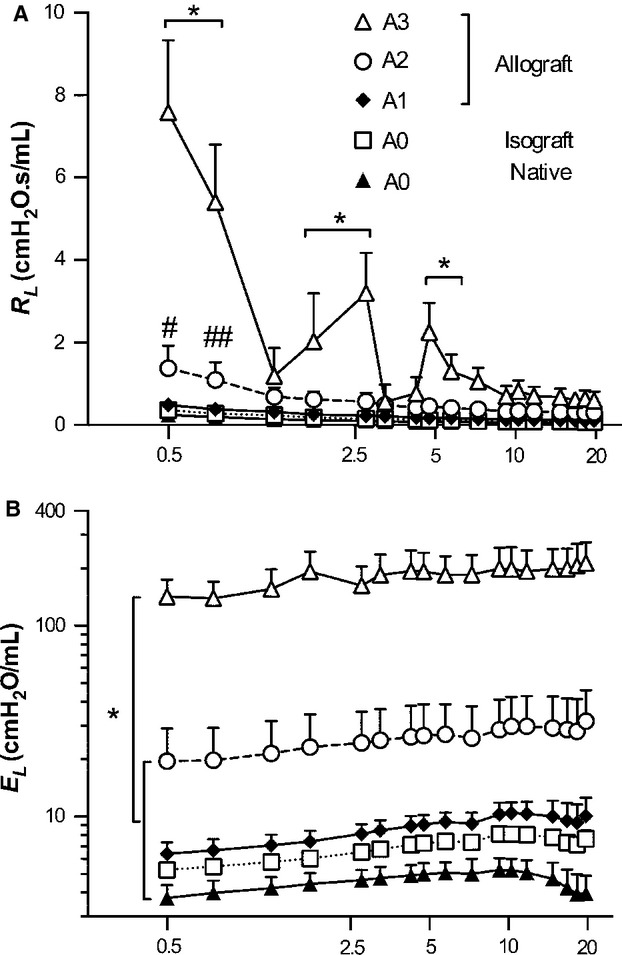
Mean ± SD of *R*_L_ and *E*_L_ at 4 cmH_2_O of PEEP level. (A) *R*_L_ was significantly dependent on both frequencies (*P *< 0.0001) and groups (*P *< 0.0001), and there was an interaction between them (*P *< 0.0001). A3 was increased than the other groups below 7.5 Hz (*P < 0.001). Statistical significances between A2 and A1 were denoted by # and ##; *P *< 0.01 and *P *< 0.05, respectively. (B) *E*_L_ was also significantly depended on groups (*P *< 0.0001), and A3 was absolutely increased than the other groups in all over the frequencies (*P < 0.001). *E*_L_ in A2 was continuously increased. Peak value of native control (A0), isograft (A0), and A1 groups appeared under 10 Hz, and then decreased gradually.

The experimentally measured values of *R*_L_ and *E*_L_ at 4 cmH_2_O of PEEP are shown in Figure 2. Both *R*_L_ and *E*_L_ were significantly dependent on the frequency (*P *< 0.0001), groups (*P *< 0.0001), and there was a significant interaction between them (*P *< 0.0001).

The data from A3 were quite distinct. *R*_L_ in A3 was significantly higher than in the other groups below 7.5 Hz (Fig. 2A, *P *< 0.001) On the other hand, A2 was higher than A1 only at low frequencies including at 0.5 Hz (1.76 ± 0.87 vs. 0.84 ± 0.49 cmH_2_O.s/mL, *P *< 0.01), and 0.75 Hz (1.38 ± 0.49 vs. 0.67 ± 0.38 cmH_2_O.s/mL, *P *< 0.05). *E*_L_ in A3 was strongly elevated at all frequencies compared to all other groups (Fig. 2B, *P *< 0.001). *E*_L_ in A2 was almost three times higher than in A1 (20.9 ± 9.4, and 6.4 ± 0.9 cmH_2_O/mL, respectively). Interestingly, the maximum value of *E*_L_ (*E*_max_) in the A0, A1, and A2 groups appeared under 10 Hz, whereas A3 showed a frequency‐dependent increase. In the normal lung, at higher frequencies, airway inertance becomes dominant, and dynamic *E*_L_ shows a decrease (Kaczka et al. 1997). The low‐frequency features should be sensitive to tissue stiffness (*E*_L_ < 2 Hz), and airway closure, alveolar flooding/collapse, and derecruitment (Mead 1969; Kaczka et al. 2001; Lutchen et al. 2001; Bellardine et al. 2005). These results suggested that there were massive alveolar and airway closure/flooding, and increased tissue stiffness in A3.

**Figure 3.**
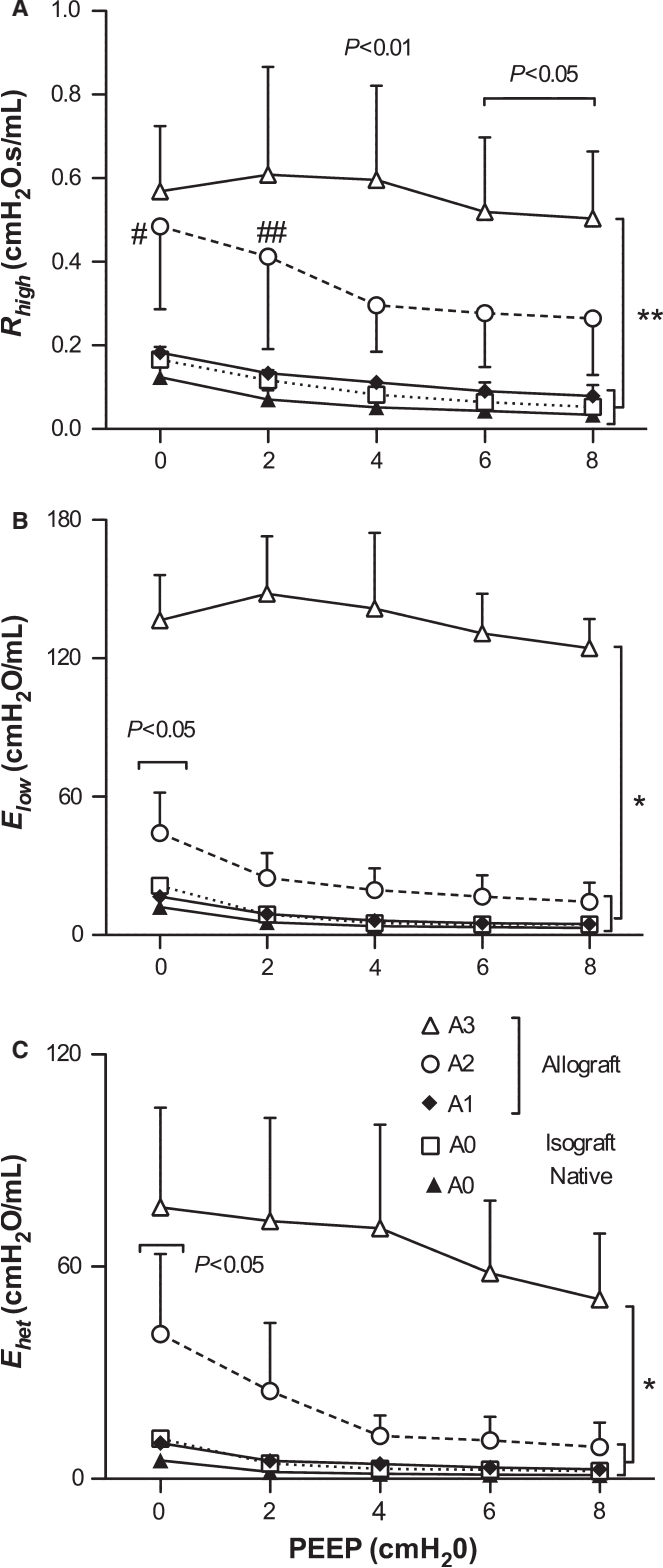
Mean ± SD of *R*_high_, *E*_low_, and *E*_het_ as function of PEEP levels. (A) *R*_high_ in A3 was increased than both A1 and A0 at all PEEP level (**P < 0.001), and also higher than A2 above PEEP 4 cmH_2_O (*P *< 0.01 and *P *< 0.05, respectively). Significance denoted by # and ##; between A2 and A1 (*P *< 0.01 and *P *< 0.05, respectively). (B) *E*_low_ of A3 was increased than the other group in all PEEP level (*P < 0.001), and A2 was only increased than A1 and A0 at PEEP 0 cmH_2_O (*P *< 0.05). (C) All group of *E*_het_ were decreased according to PEEP levels. A3 was increased than the other group in all PEEP level (*P < 0.001), and A2 was increased than A1 and A0 at PEEP 0 cmH_2_O (*P *< 0.05).

To investigate the effect of PEEP, we evaluated the *R*_high_, *E*_low_ (*E*_L_ at 0.5 Hz), and *E*_het_ (*E*_max_ − *E*_low_) with changing PEEP levels between 0 and 8 cmH_2_O (Fig. 3). *R*_high_ (Fig. 3A) represents mostly airway resistance (Bellardine et al. 2005). *R*_high_ in A3 was significantly higher compared to A0 and A1 at all PEEP levels (Fig. 3A, *P *< 0.001), and increased more than A2 at 4 cmH_2_O or higher PEEP levels (*P *< 0.01, and *P *< 0.05, respectively). In A2, *R*_high_ was higher than in A1 at lower PEEP levels: at 0 cmH_2_O (0.48 ± 0.20 vs. 0.18 ± 0.01 cmH_2_O.s/ml, *P *< 0.01), and 2 cmH_2_O (0.41 ± 0.22 vs. 0.13 ± 0.02 cmH_2_O/ml, *P *< 0.05). Intriguingly, A2 and the lower A‐grade groups showed a decrease in *R*_high_ with increasing PEEP and then plateaued at PEEP 4, although the impact of PEEP in A3 was unclear.

*E*_low_ (Fig. 3B) in A2 was higher than in A1 only at 0 cmH_2_O PEEP (44.2 ± 17.7 vs. 16.7 ± 1.5 cmH_2_O/ml, *P *< 0.05), and decreased with the influence of PEEP. In A3, *E*_low_ was substantially higher than in the other groups at all PEEP levels. *E*_low_ in A3 did not seem to be affected by PEEP at low PEEP levels, but gradually decreased at high PEEP levels.

*E*_het_ (Fig. 3C) reflects the frequency dependence of lung elastance which is related to structural heterogeneity of the disease (Lutchen et al. 2001; Bellardine et al. 2005, 2006). In A2, *E*_het_ was higher than in A1 at 0 cmH_2_O PEEP (40.9 ± 22.7 vs. 10.2 ± 0.9 cmH_2_O/ml, *P *< 0.05), and showed the same pattern as *E*_low_ and *R*_high_ with increasing PEEP. In A3, it is notable that *E*_het_ continuously improved with PEEP, at least until PEEP 8 cmH_2_O. Thus, the A2 lung was sensitive to PEEP. On the other hand, the A3 lung did not show signs of recruitment in our PEEP range.

**Figure 4.**
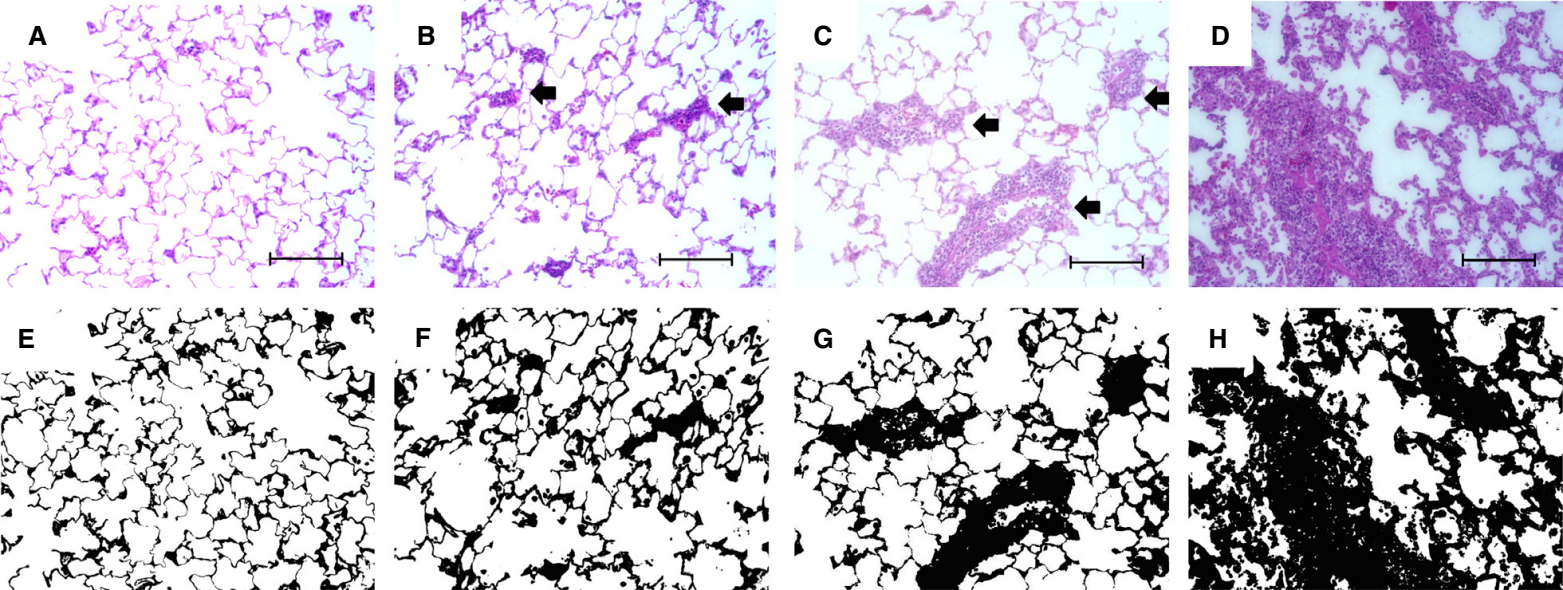
Examples of hematoxylin and eosin‐stained sections of lungs after transplantation. (A) A0 normal lung, (B) A1 lung showed a sparse perivascular mononuclear infiltrates, (C) A2 lung had more frequent and wide perivascular infiltrates, which were readily recognized at low magnification. (D) A3 showed extensive infiltration not only in perivascular and peribronchiolar, but also into interstitial and alveolar airspace. (E–H): binary images that were converted from (A–D). Tissue fraction ratio (TF) was calculated from the ratio of tissue and cell infiltration areas (black) to the total area (black + white). Original magnification was ×20. Bar represents 100 *μ*m. Arrows showed perivascular mononuclear infiltrate lesions.

Representative A‐grade histopathological sections are shown in Figure 4. Isograft lung had no rejection (Fig. 4A). In allograft lung, A1 shows spare perivascular mononuclear infiltrates (Fig. 4B), whereas A2 has more frequent and larger infiltrate regions (Fig. 4C). The appearance of A3 (Fig. 4D) had changed compared to A1 and A2, because the extent of infiltration is not only in perivascular and peribronchiolar, but also in alveolar septum. Figure 4E–H are the binary images that were converted from Figure 4A–D.

**Figure 5.**
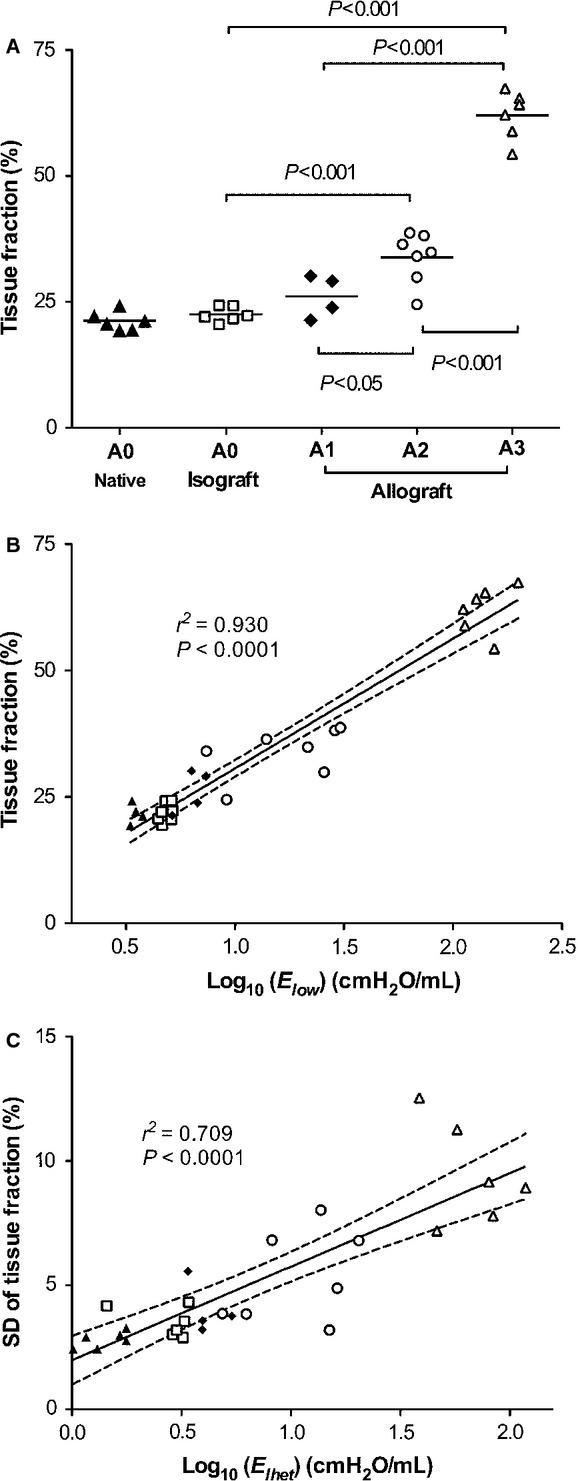
(A) Mean value of TF (%) with individual animals. Horizontal bar indicates mean value of each group. (B) Relationship between TF (%) and *E*_low_ (log scale) showed the strong correlation *(r*^2^ = 0.930, *P *< 0.0001), and (C) SD of TF (%) and *E*_het_ (log scale) indicated the significant correlation *(r*^2^ = 0.709, *P *< 0.0001). Black line showed the linear regression, and dot lines were 95% confidence intervals.

Figure 5A shows the mean and SD value of TF (%) for each animal. Although there was no difference between the mean TF of isograft and A1 (22.5 ± 1.5, and 26.1 ± 4.2%, respectively), TF in A2 (33.8 ± 5.1%) was higher than in A1 (*P *< 0.05). Furthermore, TF in A3 significantly increased (62.1 ± 4.7%) compared to that in A2 (*P *< 0.001). From these results, we suggested that TF would be good index to quantify A‐grade. The relationship between TF (%) and *E*_low_ (log scale) showed a strong correlation (*r*^2^ = 0.930, *P *< 0.0001) as seen in Figure 5B. TF reflects the pathological A‐grade, and this index strongly correlated with *E*_low_. Furthermore, SD of TF (in each animal) and *E*_het_ (log scale) also indicated a significant correlation (*r*^2^ = 0.709, *P *< 0.0001) in Figure 5C. Thus, we quantified A‐grade with TF, and found that TF related to both *E*_low_ and *E*_het_.

**Figure 6.**
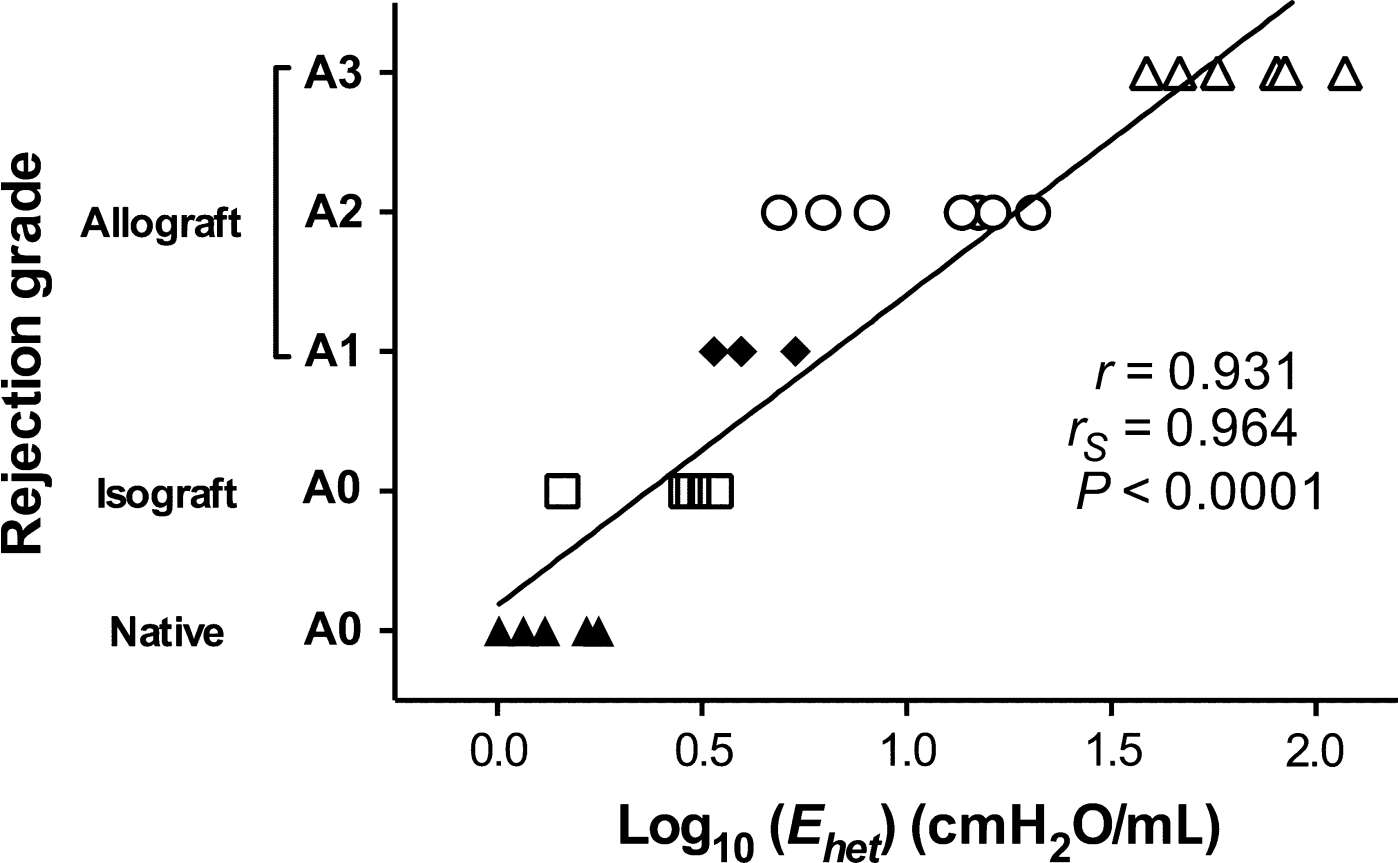
Plots of A‐grade and *E*_het_ (log scale) at PEEP 4 cmH_2_O in individual animals. Pearson correlation coefficient (*r*) was 0.931, and Spearman's rank correlation coefficient testing (*r*_*s*_) was 0.964 (*P *< 0.0001, respectively). Black line showed the regression of Pearson correlation.

Next, we assessed the correlation between AR grade and the frequency‐dependent properties of lung impedance as described in hypothesis. Figure 6 shows A‐grade and *E*_het_ at PEEP of 4 cmH_2_O. Pearson's correlation coefficient and Spearman's correlation coefficients showed the strong correlation (*r* = 0.931, *r*_*s*_ = 0.964, *P *< 0.0001, respectively), suggesting that *E*_het_ predict A‐grade in a rat lung transplantation model.

## Discussion

The purpose of this study was to investigate changes in airway and tissue mechanical properties in a rat model of AR. In particular, to evaluate the progression of AR, the histological A‐grade classification was used in accordance with the International Society for Heart and Lung Transplantation guidelines. Our study produced three main results, (1) *R*_L_ and *E*_L_ corresponded with the histopathological A‐grade, and (2) A‐grade can be quantified with TF as demonstrated by the excellent correlation between TF and *E*_low_, and SD of TF and *E*_het_. Also, *E*_het_ appears to be a good index to noninvasively estimate A‐grade.

There are several studies that reported that the traditional spirometric indices such as FEV_1_ decreased during AR after lung transplantation in human patients (Otulana et al. 1990; Van Muylem et al. 1997). However, FEV_1_ and other indices derived from forced maximum flow‐volume maneuvers are influenced by wound pain, fatigue, and the other factors (Mead 1979), especially in perioperative patients (Cooper 2011) and preschool children (Beydon et al. 2007). Furthermore, in patients during mechanical ventilation the spirometric tests cannot be applied. Additionally, lung impedance measurements are sensitive to changes in specific structures in the lung manifesting as unique signature changes when examined over a particular frequency range (Lutchen and Gillis 1997). These changes in frequency characteristics of impedance can provide insight into the alterations in structure (Bellardine Black et al. 2007) such as alveolar wall destruction in emphysema or air space fluid filling in lung injury with altered regional tissue compliances (Bellardine et al. 2005; Ito et al. 2005). Goldman et al. (Goldman et al. 2005) followed up the lung impedance of the patient after lung transplantation, and found that the real and imaginary parts of impedance were increased by AR with biopsy evident, without change in spirometric index. Thus, lung impedance measurements have the potential to complement the conventional spirometric data and avoid TBLB.

The baseline frequency dependence of *R*_L_ and *E*_L_ from 0.1 to 1.0 Hz is primarily due to the viscoelastic properties of lung tissue (Lutchen and Suki 1996). This includes the frequency‐dependent drop in baseline *R*_L_ from 0.1 approaching a constant at 1 Hz representing the sum of airway and chest wall resistance and the slight frequency‐dependent increase over the same frequency range followed by a decrease as the airway inertia becomes more dominant. The increase in *E*_low_ (Fig. 2B) can result from several factors including the increase in alveolar wall stiffness, increase in surface tension, and small airway and airspace closure. Specifically, *E*_low_ in A3 was substantially elevated because of extensive cellular infiltration not only in perivascular and peribronchiolar area, but also in alveolar walls (Fig. 4D and 4H). Hence, we believe the main reason for the high *E*_low_ is surface film stiffening and alveolar collapse. This pathogenesis was also reflected in TF. Indeed the mean value of TF in A3 (Fig. 5A) increased by 83% compared to A2, and *E*_low_ in A3 increased by nearly 7‐fold compared to its value in A2. In A2, the mean TF also increased by 29%, and by 3‐times compared to its value in A1 (Fig. 2B). Thus, the simultaneous increases in TF and *E*_low_ resulted in a good correlation between these two variables (Fig. 5B). Consequently, TF reflects the altered structural nature of AR during the progression of the disease, and *E*_low_ allows us to infer the pathological alterations in structure.

The strategy of mechanical ventilation management for AR is still debated, because there was no clinical trials and PEEP study for AR yet. In animal model, increased peak airway pressure was reported during ventilation in AR (Jung et al. 2006; Liu et al. 2006). However, these studies did not investigate PEEP effect. Therefore, many cases were treated according to the ALI/ARDS guidelines, especially PEEP management. We assessed the impact of PEEP using the frequency‐dependent indexes of dynamic lung mechanics of AR (Fig. 3). In a recent study, Malbouisson et al. (Malbouisson et al. 2001) demonstrated that an increase in gas volume with PEEP throughout the lung depends on the difference in regional compliance, implying that PEEP should be set in a way to homogenize the distribution of regional lung compliances. In ALI/ARDS, features of quasi‐static respiratory mechanics such as the inflection points along the quasi‐static pressure‐volume curves are thought to allow the choice of the optimal PEEP level to prevent alveolar overdistention injuries and to minimize cyclic reopening of collapsed alveoli during mechanical ventilation (Amato et al. 1995). However, ventilation is superimposed on the dynamic properties of the lung, and hence the dynamic mechanical properties may provide different information from static measurements. Recent studies have reported the absence of the lower inflection point in the pressure‐volume curve and have attributed this to the heterogeneous nature of the lung disease (Vieira et al. 1999; Maggiore et al. 2003). Moreover, Bellardine Black et al. (2007) showed that quasi‐static lung mechanics did not reflect regional lung compliance in an ARDS animal model. In a heterogeneously collapsed lung, increases in PEEP will recruit airspaces while simultaneously overdistending normal regions. In contrast, the frequency dependence of dynamic respiratory mechanics was more sensitive to such regional inequality (Bellardine et al. 2005; Kaczka et al. 2005, 2009; Bellardine Black et al. 2007). The ability to quantify mechanical heterogeneity under dynamic conditions may have potential for optimizing ventilatory parameters such as PEEP, tidal volume, or frequency (Kaczka et al. 2005). In our study, it appeared that *E*_het_ (Fig. 3C) exhibited a negative PEEP dependence in all group suggesting the heterogeneity of disease in AR that is reduced by increasing the PEEP.

PEEP effect was carried out in order to evaluate whether such mechanisms as recruitment can also influence the rate of tissue distortion or heterogeneity. Furthermore, plots of A‐grade and *E*_het_ (Fig. 6) showed that A‐grade can be estimated from *E*_het_ and *E*_low_, suggesting that both mechanics measurement indexes are useful in predicting A‐grade. As stated above, measurement of lung impedance at multiple frequencies is sensitive to changes in the frequency‐dependent features of lung mechanics and enables the assessment and monitoring of AR after lung transplantation. This information would provide insights into the pathophysiology of lung diseases and could be valuable in the design of effective treatment protocols.

Clinically, the most important differential diagnosis from AR is infections. Nonspecific nature of symptoms, signs, and conventional laboratory findings are often inadequate for diagnosis of AR (Penketh et al. 1988). Regarding pulmonary function, some studies reported that AR is usually accompanied with airflow obstruction, but infections produce a restrictive impairment or an isolated decrease in diffusing capacity (Penketh et al. 1988; Otulana et al. 1990). Van Muylem et al. (1995, 1997) also showed a rise in the slope of the alveolar plateau during inert gas washout, and both infection and rejection were accompanied by airflow obstruction. Recently respiratory impedance of patients in pneumonia were measured by FOT (Lorx et al. 2010), and applied various model fitting to these data to assess the altered tissue and airway properties. We choose not to fit our data with model; rather, we directly extracted the frequency‐dependent features of impedance. Nevertheless, fitting advanced impedance models would be useful to characterize the mechanical properties of the lung and potentially distinguish between AR and infection.

This study has several limitations. First, we analyzed the mechanics of one‐lung only under the open‐chest condition after euthanasia in order to prevent artifacts of single lung ventilation such as pulmonary edema or ALI/ARDS as much as possible, especially for A3 lungs. However, several images of A3 also showed some alveolar fluid, which was counted as tissue and hence was included in the correlation with elastance. Various factors including lung volume changes, the effect of the chest walls, loss of blood, and hemorrhage shock may have had an impact on lung impedance (Hirai et al. 1999; Peták et al. 2002; Sly et al. 2003). However, there is a report that lung impedance at low frequencies were no difference between in vivo with open‐chest condition and in isolated lung (Suki et al. 1991). Second, we could not apply FOT in the A4 lungs because of the excessively high airway pressures. Also, we could not apply a PEEP higher than 8 cmH_2_O in A3 animals. Third, in Figure 2, the impedance curves of A3 was fluctuating, especially in *R*_L_ (Fig. 2A), suggesting strong nonlinear harmonic distortion because of the spectrum of the pseudorandom noise signal in flexiVent contains noninteger multiples of each others (Suki and Lutchen 1992). Last, we did not assess small airway rejection (B‐grade) which is also a risk factor for BOS (Davis et al. 2012). Early after allogeneic hematopoietic stem‐cell transplantation, Barisione et al. (Barisione et al. 2012) founded an increased airway distensibility with lung inflation probably due to an enhanced airway‐to‐parenchyma coupling. In our data, *R* in A3 did not change with different PEEP levels (Fig. 3A) whereas in A2 and A1 lungs, *R* values were decreased with PEEP, suggesting that the A1 and A2 lungs might increase coupling of airways to lung parenchyma, but not the A3 lungs. We also did not measure absolute lung volume, which would be helpful to investigate the relationship between airway and parenchymal properties in future study.

In this study, we found that FOT could be used to noninvasively characterize lung function in AR. Our findings from the FOT measurements were closely related to the altered lung pathology evaluated from more traditional methods using histopathological sections. These results suggest that FOT could complement standard clinical assessment for a more rapid recognition of AR due to the noninvasive nature and the simplicity with which pulmonary mechanics can be measured using FOT. Nevertheless, further studies are necessary to characterize the sensitivity and specificity of the indexes derived from lung mechanics in AR. This study is the first to report lung impedance data in a rat AR model. While more work is required before these results can be translated to human subjects, this study illustrates the potential of using mechanics measurements as a noninvasive tool for vascular and airway rejection in the clinic.

## Acknowledgment

We thank M. Kurozumi, Department of Clinical Laboratory, Kyoto University Hospital, for classifying pathology.

## Conflict of Interest

None declared.
